# Near-Infrared Reflective Polymer Films Based on UV-327-Doped Zinc Oxide Nanoparticles

**DOI:** 10.3390/ma16247660

**Published:** 2023-12-15

**Authors:** Xiaohui Zhao, Yutong Liu, Yue Cao, Hui Cao, Huihui Wang, Zhou Yang, Dong Wang, Wanli He

**Affiliations:** 1 School of Materials Science and Engineering, University of Science and Technology Beijing, Beijing 100083, China; m202120498@xs.ustb.edu.cn (X.Z.); lytong99@163.com (Y.L.); m202210273@xs.ustb.edu.cn (Y.C.); yangz@ustb.edu.cn (Z.Y.); wangdong@ustb.edu.cn (D.W.); hewanli@mater.ustb.edu.cn (W.H.); 2 Key Laboratory of Optoelectronics Technology, Ministry of Education, Beijing University of Technology, Beijing 100124, China

**Keywords:** cholesteric liquid crystal, pitch gradient distribution, broadband reflection, UV-327, zinc oxide nanoparticles

## Abstract

We prepared cholesteric liquid crystal (CLC) films with broadband reflective properties by admixing organic dye UV-327 into inorganic zinc oxide nanoparticles (ZnO NPs), utilizing the principle of pitch distribution from a large to a small gradient along the film thickness direction, leading to broadband reflection. ZnO NPs are poorly dispersed and easy to gather, but they do not decompose easily. The addition of UV-327 makes up for the above shortcomings. UV-327 is an organic compound with good compatibility and dispersion with liquid crystal systems. Therefore, we used the method of mixing two UV-absorbing dyes (UV-327 and ZnO NPs) to obtain CLC films. UV-absorbing dyes (UV-327 and ZnO NPs) made the liquid crystal films form a UV intensity gradient in the direction of thickness, prompting the polymerizable monomers to polymerize faster on the stronger side of the light, leading to the relative diffusion of chiral molecules and polymerizable monomers, forming the concentration gradient of chiral molecules in the direction of thickness. The pitch has a gradient distribution as the chiral concentration varies. Then, anchored by the polymer network, the pitch gradient distribution no longer changes. Broadened reflective bandwidth can reach up to 881 nm. Furthermore, the film covers the near-infrared wavelength band well, which can be applied to future smart windows or laser shielding for medical and military applications. It is also believed that this achievement will optimize the preparation technology of broadband reflective CLC films in the future.

## 1. Introduction

Cholesteric liquid crystals (CLCs) exhibit distinct and remarkable optical characteristics [1,2,3,4,5,6], including selective reflection, optical effects, and circular dichroism. Selective reflection refers to the ability of cholesteric liquid crystals to reflect specific light wavelengths [7,8,9]. This phenomenon is governed by Bragg’s Law [10,11,12,13]. Along the direction of the helical axis, the CLCs selectively reflect incident light. It follows from Bragg’s formula that the central reflection wavelength of CLC is denoted as λ = nP, where n represents the CLC average value of the ordinary refractivity (n_o_) and the extraordinary refractivity (n_e_) of the local monoaxial structure, and P represents the pitch of CLCs. The reflected bandwidth of CLC is expressed as ∆λ = ∆nP, with ∆n being the birefringence (∆n = n_e_ − n_o_). Within this reflected bandwidth range, left-handed CLC allows right-handed polarized light to pass through while reflecting left-handed polarized light [14,15,16,17]. Beyond the reflected bandwidth range, both left-handed and right-handed polarized light can pass through the left-handed CLC.

However, the narrow reflectance bandwidth of the single-pitch CLC leads to various limitations. Realizing high ∆n in CLC faces challenges such as complex synthesis and poor stability [18]. It is therefore difficult to adjust ∆λ by easily increasing ∆n. This approach involves the doping of different concentrations and types of chiral compounds, which have different effects on the pitch (P) of the cholesteric liquid crystals. The equation describing this relationship is P = [(HTP) × c]^−1^, where HTP denotes the helical twisting force of the chiral compound, which is related to the type of chiral compound, and c denotes the content of the chiral compound. Many theoretical studies and experimental methods have proved that the only way to realize the broadband reflection of cholesteric liquid crystals is to start from the pitch gradient distribution or non-uniform pitch distribution [19].

Kralik has succeeded in stacking three layers of CLC films with selectively reflective wavelengths in the red, green, and blue light bands, respectively, realizing the non-uniform arrangement of pitches in the thickness direction, and preparing a film that selectively reflects the entire visible light region [20]. Broer used photopolymerization to make a CLC film by combining a complex of diacrylates, monoacrylates, dyes, nematic liquid crystals, and photoinitiators [21,22]. The presence of UV-absorbing dyes creates a UV intensity gradient from the top (near the light source) to the bottom (away from the light source) in the direction of the liquid crystal film thickness when illuminated with UV light. Since the consumption rate of bifunctional polymerizable monomer molecules is faster than that of monofunctional polymerizable monomers during the photoreaction process, the bifunctional monomers polymerize quickly and tend to diffuse upward to the top surface, while the monofunctional monomers polymerize slowly and tend to diffuse downward to the bottom surface. The concentration of bifunctional chiral monomers near the upper surface is higher, the pitch of molecular arrangement is smaller, and the opposite is true near the lower surface [23]. Yang used chiral compounds whose helical twisting force increases with increasing temperature to prepare polymer-stabilized cholesteric liquid crystal films whose reflective bandwidth broadens with increasing temperature by triggering cross-linking reactions between monomer molecules in a liquid crystalline polymerizable monomer/nematic liquid crystal/chiral compound composite system [24]. In recent years, Yang has prepared chiral azobenzene compounds with photoresponsive properties instead of thermoresponsive chiral compounds and once again succeeded in preparing CLC films that can undergo broadband reflections [25]. In addition to this, the non-uniform distribution of pitch can be induced by electric or magnetic fields. White reports substantial (over 1500 nm) and reversible reflection trap tuning of polymer-stabilized cholesteric liquid crystals (PSCLCs) when a direct current (DC) electric field is applied. A selective and reproducible tuning range from 100 nm to 400 nm is demonstrated. Responsive chiral molecules added to the liquid crystal system move according to external factors, and their concentration is altered to achieve a pitch gradient distribution [26]. Cao et al. succeeded in preparing polymer-stabilized cholesteric liquid crystal films with broadband reflectivity using a three-layer self-diffusion system, where the polymer network of the bilayer PSCLCs itself was used as a diffusion channel. Chiral compounds and UV-absorbing dyes were diffused from the cholesteric liquid crystal layer to the nematic liquid crystal layer by the diffusion gradient method, controlling the contents of R5011 and UV-327 and adjusting λm and Δλ to obtain liquid crystal films capable of broadband reflection [27].

Nowadays, there are many studies on the doping of metal nanoparticles in cholesteric liquid crystal films. Zhu et al. doped ATO nanoparticles in PSLC films [28]. The transmittance of the films can be reversibly varied between 78.5% and 10% in the visible range, shielding up to 80.7% of the infrared radiation without affecting the visible transmittance. The ATO nanoparticles also reduced the threshold voltage and optimized the response performance of PSLC. Wang et al. prepared near-infrared broadband reflective polymer-stabilized cholesteric liquid crystal (PSCLC) films by spin-coating with MoO_2_ [29]. The reflective broadband of the PSCLC films broadened from 261 nm to 401 nm. It was confirmed that the nanoparticles have the ability to undergo photo-thermal conversion and that the diffusion of the nanoparticles in the liquid crystal composite system is conducive to the formation of non-uniform spacing distributions. Zhao et al., proposed a new method to prepare broadband reflective films using nanofibers loaded with zinc oxide nanoparticles as network supports in chiral nematic liquid crystals, achieving a broadening of the visible range from 246 nm to 412 nm. The films demonstrated excellent UV shielding and IR thermal control effects, providing a potential method for the preparation of future smart windows [30].

In addition to this, doped dyes can be studied for a wide range of applications related to hyper-reflective smart windows and laser emitters. Khandelwal et al. have prepared cholesteric-phase liquid crystal films that can reflect more than 60% of the total energy of solar infrared radiation while possessing high transmittance. Simulation of this film over a typical building window would have a significant impact on the indoor temperature of living and working spaces [31]. Lucchetta et al. realized a DFB laser based on a dye-doped holographic reflective grating embedded in a PMP film. The PMP mixture is placed around the grating dots, and the curing/photopolymerization process is subsequently facilitated by UV irradiation. This process results in a PMP-DFB system that can produce laser action [32].

In our previous studies, the effects of the UV-absorbing dyes UV-327 and ZnO NPs on broadband reflective films were investigated, respectively [33,34]. The impact of UV absorber concentration, UV irradiance, irradiation time, polymerization temperature, and concentration of polymerizable monomer C6M on the reflected bandwidth of the samples was discussed. The experimental results show that the broadest reflective band of the films containing ZnO NPs can reach 706 nm, and the broadest reflective band of the films containing UV-327 can reach 616 nm. However, ZnO NPs are very stable UV-absorbing dyes, but they cannot be uniformly dispersed in liquid crystal systems. The UV absorption efficiency is not as good as that of benzotriazoles UV-327; while UV-327 cannot be irradiated under UV light for a long time, it is easy to decompose, as well as under heating conditions. However, the dispersion of UV-327 in the liquid crystal system is much better than that of the former because of the principle of similar solubility. So by doping UV-327 in ZnO NPs, we can optimize the effect of metal nanoparticles when used in UV-absorbing dyes.

Therefore, we perfected the technique of preparing broadband reflective CLC films containing UV-absorbing dyes to improve the above-mentioned drawbacks. In this research, a mixed system of organic and inorganic dyes was directly selected to compensate for both the poor dispersion of inorganic dyes and the instability of organic dyes. R5011 was chosen as the chiral compound because of its larger HTP value and the smaller amount added to achieve the predetermined effect. RM257 was chosen as the polymerizable monomer that can be rapidly polymerized to obtain a polymer network for the fixation of the formed pitch. The two dyes synergized to form a light intensity gradient in the direction of the liquid crystal film thickness under low-intensity UV irradiation, and the polymerizable monomer RM257 had a fast polymerization rate on the light intensity side, leading to upward movement of the monomer, thus downward movement of the chiral compound R5011, which resulted in a pitch gradient, and then immobilized in a polymer network, resulting in the broadband reflective films of CLCs. The results of this study are mainly related to the near-infrared region, and the main application in real life is to apply the film to the windows of buildings to obtain infrared-adjustable smart windows. It effectively reduces the indoor temperature and the frequency of air-conditioning use in the summer, resulting in energy savings and emission reductions.

## 2. Materials and Methods

### 2.1. Materials

**Figure 1:**
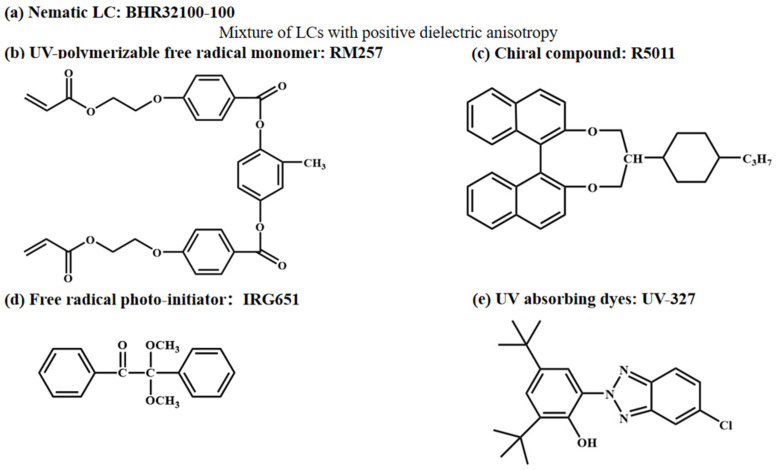
The chemical structure of experimental materials.

Figure 1 illustrates the chemical structures of the materials employed in this study. The nematic liquid crystal (BHR32100-100, n_e_ = 1.752, n_o_ = 1.517, Δn = (n_e_ − n_o_) = 0.235, Beijing Bayi Space LCD Technology Co., Ltd. Beijing, China), the chiral dopant (R5011, Shijiazhuang Chengzhi Yonghua Display Material Co., Ltd. Shijiazhuang, China), the free radical photoinitiator (IRG651, Aladdin Co., Ltd. Shanghai, China), the organic UV-absorption dye (UV-327, Annaiji Chemical Reagent Co., Ltd. Shanghai, China), the inorganic UV-absorption dye (ZnO nanoparticles, Shanghai Yien Chemical Technology Co., Ltd. Shanghai, China), and the UV polymerizable monomer (RM257, Beijing Bayi Space LCD Technology Co., Ltd. Beijing, China) were used in this study.

### 2.2. Measurements

The transmission spectrum of the samples was measured by the ultraviolet-visible-near-infrared spectrophotometer (JASCO-V570), and the transmittance of the blank cells was standardized to 100%. In general, Δλ is defined as the bandwidth at half the height of the transmitted light peak. The distribution of ZnO NPs in films was studied by polarized light microscopy (POM, Olympus BX51, Tokyo, Japan). The morphology of the pitch gradient distribution on the cross-section of the films was studied using scanning electron microscopy (SEM, Zeiss-SUPRA55, Jeol Co., Ltd., Tokyo, Japan).

### 2.3. Preparation of Samples and Cells

Cutting and cleaning of glass substrates: The indium tin oxide conductive glass was cut with the glass cutter to obtain small pieces of glass measuring 2 × 3 cm. Then, the cut glasses were cleaned into the water with detergent and put into the ultrasonic machine for 0.5 h, and after that, the new detergent water was repeated 3 times. The detergent water was then replaced with deionized water and anhydrous ethanol in turn, and the glass pieces were ultrasonicated for 0.5 h after cleaning and then removed and placed in the oven at 60 °C for drying.

Orientation processing of glass substrates: preparation of a 3 wt% PVA aqueous solution; the PVA aqueous solution was uniformly spin-coated onto the above-treated small glasses using the benchtop homogenizer; and the benchtop homogenizer was spun-coated at a speed of 3000 r/min for 30 s. The coated glasses were removed and dried in an oven at 90 °C for 2 h. The spin-coated side of the aqueous PVA solution was gently wiped with a clean flannel to obtain the parallel orientation.

Assembling liquid crystal cassettes: Parallel-oriented glass is assembled, and the two glass substrates are separated by a 20 μm thick PET spacer, which is then glued along the spacer, leaving the inlet for liquid crystal filling.

Using the electronic scale, each experimental material was weighed according to the group allocation ratios shown in Table 1 and added to the centrifuge tubes individually numbered. Subsequently, 1 mL of organic solvent, ethyl acetate, was added to the CLC mixtures and shaken well for 1 min. The centrifuge tubes were sonicated in a sonicator for an hour, removed, and placed in the oven at 70 °C for 8 h to evaporate the organic solvent. Then, remove the centrifuge tubes and wait for the samples to cool to room temperature. All of the above processes should be avoided in the light. Utilizing the capillary effect, the prepared sample is dipped into the liquid crystal cartridge with the end of a curved pin and poured into the liquid crystal cartridge through the reserved liquid crystal filling port. Afterward, the reaction is cured at 40 °C under 365 nm UV light.

## 3. Results

### 3.1. Mechanisms of the Broadband Reflection Phenomenon

**Figure 2:**
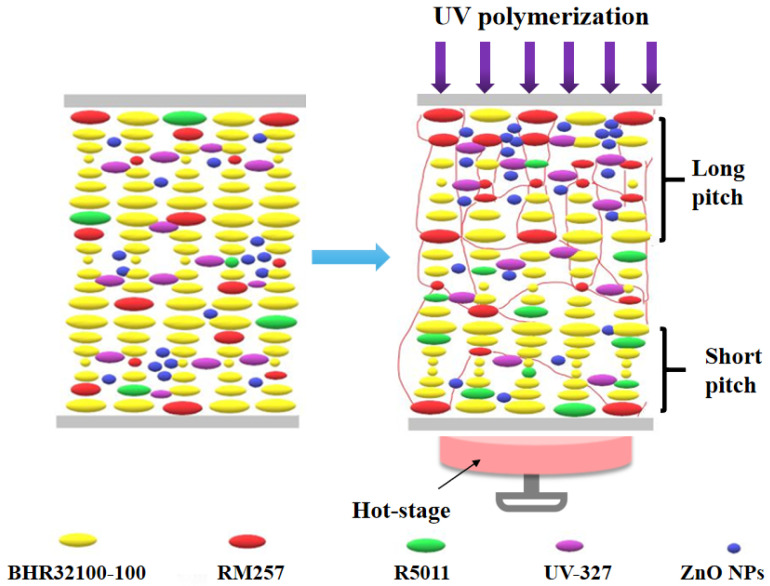
Schematic diagram of the mechanism for broadening the reflection bandwidth.

Figure 2 shows the mechanism for fabricating broadband reflective cholesteric liquid crystal films. Liquid crystal mixtures containing RM257, BHR32100-100, R5011, IRG651, and UV-absorbing dyes (UV-327 and ZnO NPs) were filled into liquid crystal cassettes and placed under low-intensity UV irradiation for polymerization. As in Figure 2, the pitch of the cholesteric liquid crystals is uniform before polymerization. Under UV irradiation, due to the presence of UV dyes, the UV intensity will be distributed in a gradient along the film thickness, and the upper UV intensity is greater than the lower UV intensity. Therefore, the polymerizable acrylate monomer RM257 has a faster rate of polymerization on the near-light side, which will promote RM257 to perform the upward diffusion motion. On the contrary, the chiral compound R5011 makes the downward movement, resulting in a larger concentration of chiral compounds on the far-light side than on the near-light side, and according to P = 1/[(HTP) × c]^−1^, the chiral compound species is determined, and the size of the cholesteric liquid crystal pitch is inversely proportional to the concentration of chiral compounds so that the cholesteric liquid crystal pitch is distributed in a gradient. The state where the upper near-light side has a large pitch and the far-light side has a small pitch is eventually formed. We used two mixes of UV-absorbing dyes here. Because UV-327 is organic, it can be well soluble in liquid crystal mixtures, and its incorporation improves the disadvantage of poor dispersion and low solubility of ZnO NPs very well. Based on the experimental results, it was determined that when mixed, by adjusting the ratio, it still provides good UV absorption.

### 3.2. Broadened Reflective Band Behavior of CLC Films with UV-327/ZnO NP Mixtures

**Figure 3:**
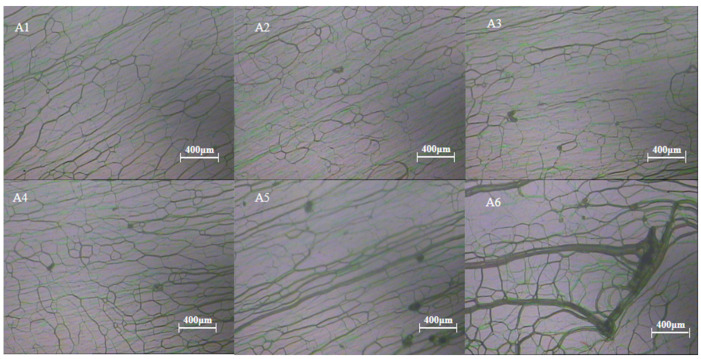
POM images of group A samples after curing.

**Figure 4:**
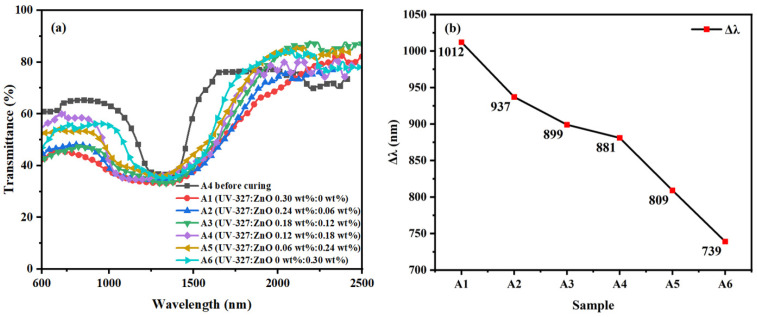
Group A samples: Trends of (**a**) transmission spectra and (**b**) Δλ with the mass fraction of UV-absorbing dyes.

Figure 3 is the polarized light microscope photograph of experimental group A after curing. Group A measured changes in the reflective bandwidth of CLC films by varying the ratio of organic and inorganic dyes while keeping the overall UV-absorbing dye content in the system fixed. We uniformly fixed the mass fraction sum of the two dyes as 0.30 wt%, followed by setting the mass fractions of UV-327 and ZnO NPs in each group as 0.30/0.00 wt%, 0.24/0.06 wt%, 0.18/0.12 wt%, 0.12/0.18 wt%, 0.06/0.24 wt%, and 0.00/0.30 wt%, respectively. The rest of the samples were grouped in proportions as shown in Table 1, and the samples obtained were named A1, A2, A3, A4, A5, and A6. Experimental group A was polymerized at 1.5 mW/cm^2^ for 20 min, and the polymerization temperature was controlled at 40 °C. As shown in Figure 3, it can be seen that the dispersion of the UV dyes deteriorates as the amount of UV-327 decreases because the nanoparticles are not soluble in the liquid crystal mixture system, so it is necessary to add soluble UV-327 to improve the dispersion of the UV-absorbing dye. As in Figure 3(A5),(A6), agglomerated ZnO NPs can be observed. The UV transmission spectra were recorded as shown in Figure 4, which records the changes in the bandwidth of each group. The reflectance bandwidth of all six groups of sample films increased significantly after polymerization compared with that before polymerization, but in Figure 4b, Δλ decreased from 1012 nm to 739 nm with the increase in the proportion of ZnO NPs. It indicates that the addition of UV-327 to ZnO NPs can effectively improve the phenomenon of poor UV absorption due to the poor dispersion of ZnO NPs, and therefore increasing the proportion of UV-327 in a liquid crystal hybrid system is beneficial to improving the UV absorption effect. But also ensure that the reflective bandwidth effect of CLC films is favorable. When the UV-absorbing dyes were adjusted in proportion, the A1–A3 and A4–A6 groups showed a larger decrease, while the A3-A4 group showed a relatively flat trend. In conjunction with the POM image, the samples in groups A3 and A4 showed little difference in dispersion. However, group A4 meets the goal of our study with ZnO NPs as the main UV-absorbing dye. Moreover, the reflective bandwidths of the A3 and A4 groups are not very different. The above analysis shows that the mixing ratio of sample A4 is appropriate with proper dispersion, and on this basis, it shows a relatively large Δλ.

### 3.3. RM257 Concentration Dependence of the Reflection Bandwidth

**Figure 5:**
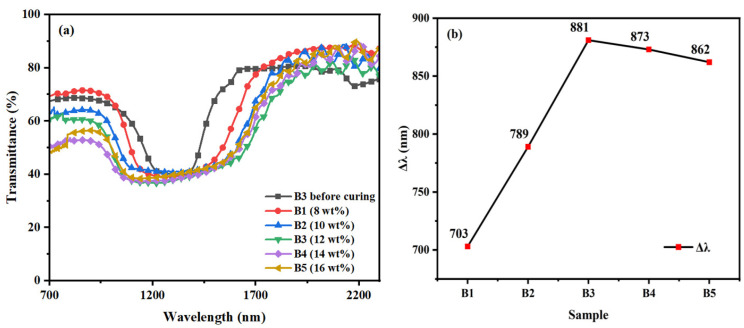
Group B samples: Trends of (**a**) transmission spectra and (**b**) Δλ with the mass fraction of RM257.

Figure 5 illustrates the transmission spectra and corresponding Δλ for the samples (B1–B5) listed in Table 1 after polymerization. Samples (B1–B5) were prepared with different mixing ratios of the polymerizable monomer RM257 as described in Table 1. These samples (B1–B5) were reacted at 40 °C for 20 min under UV irradiation at 1.5 mW/cm^2^. The results show that as the concentration of polymerizable monomer RM257 increased from 8 wt% to 12 wt%, Δλ also increased from 703 nm to 881 nm. However, when the concentration of polymerizable monomer RM257 was increased from 12 wt% to 16 wt%, Δλ decreased from 881 nm to 862 nm.

The observed tendency of Δλ to increase and then decrease with increasing content of polymerizable monomer RM257 can be explained as follows: with the control variable approach, different concentrations of polymerizable monomer RM257 samples (B1–B5) were reacted at the same UV intensity, the same temperature, and the same duration so that we could isolate the effect of the concentration of polymerizable monomer RM257 on Δλ. When the concentration of polymerizable monomer RM257 is low, the migration rate of both polymerizable monomer RM257 and chiral molecule R5011 is slow, and the density of the polymerization network formed is moderate so that the small molecules can perform migratory movement in it. As the concentration of the polymerizable monomer RM257 increases, the migration of the chiral molecule R5011 is accelerated, resulting in a rise of Δλ. On the contrary, when the concentration of the polymerizable monomer RM257 was increased from 12 wt% to 16 wt%, the rapid polymerization of the polymerizable monomer RM257 formed a denser polymerization network, which hindered the migration of the chiral molecule R5011 and led to the decrease of Δλ. The reflective bandwidth of the film is maximized when the polymerizable monomer content is 12 wt%.

### 3.4. Effects of the Chiral Compound R5011

**Figure 6:**
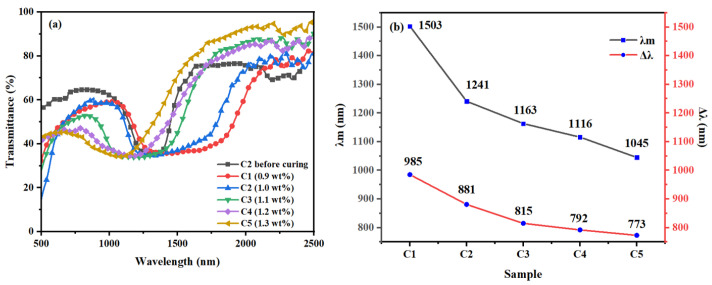
Group C samples: Trends of (**a**) transmission spectra and (**b**) Δλ with the mass fraction of R5011.

The samples (C1–C5) in Table 1 were prepared using different ratios of the chiral molecule R5011. These samples (C1–C5) were reacted at 40 °C for 20 min under UV irradiation at 1.5 mW/cm^2^. As illustrated in Figure 6, it is noteworthy that as the compositional concentration of the chiral molecule R5011 increases from 0.9 wt% to 1.3 wt%, there is a decrease in Δλ and λ, with Δλ decreasing from 985 nm to 773 nm and λ decreasing from 1503 nm to 1045 nm. According to the equation P = [(HTP) × c]^−1^, which decreases as the concentration of the chiral molecule R5011 increases, resulting in decreasing λ. The broadening effect of the formation of Δλ by diffusion is greater than the effect of narrowing the pitch due to the increase in chiral content, and Δλ shows an overall progressive decrease. However, the higher the concentration of R5011, the greater the decrease in Δλ, and the closer the center wavelength λ is to the visible band. The central wavelength of C2–C5 varies much less than that of C1–C2. Therefore, it is better to use 1 wt% of R5011, because the reflected broadband is larger and λ is also in the appropriate band.

### 3.5. The Influence of Other Factors on the Reflectance Bandwidth of Samples

#### 3.5.1. Polymerization Temperature on the Reflectance Bandwidth of Samples

**Figure 7:**
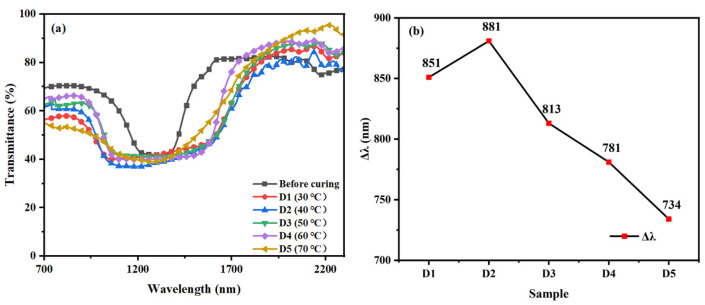
Group D samples: Trends of (**a**) transmission spectra and (**b**) Δλ with polymerization temperature.

The samples with fixed composition ratios were reacted at different temperatures (30 °C, 40 °C, 50 °C, 60 °C, and 70 °C) for 20 min at 1.5 mW/cm^2^ UV intensity according to Table 1, named D1, D2, D3, D4, and D5, respectively. As can be seen from Figure 7a, Δλ also increased from 851 nm to 881 nm when the reaction temperature was increased from 30 °C to 40 °C. In contrast, Δλ decreased from 881 nm to 734 nm when the reaction temperature was increased from 40 °C to 70 °C. The trend of Δλ increases and then decreases with increasing temperature. The polymerization temperature mainly affects the rate of molecular diffusion, with relatively low temperatures resulting in small rates of molecular diffusion and higher temperatures also resulting in too fast diffusion, both of which make it difficult to form more pronounced molecular concentration gradients. When the polymerization temperature is less than 40 °C, due to the thermal movement of molecules, the temperature increases, the UV-absorbing dye diffusion is accelerated, a larger concentration gradient is formed, and a larger UV intensity gradient in the direction of the thickness is obtained, which leads to a consequent increase in the diffusion rate of RM257 and R5011, and a larger pitch gradient will be obtained in the same range of polymerization time, which leads to a larger Δλ. When the polymerization temperature is greater than 40 °C, the temperature increases, and the polymerization rate and diffusion rate will increase accordingly, but at this moment, the polymerization rate exceeds the diffusion rate and becomes dominant. There will be a situation in which the polymerization is finished but the diffusion is not complete, resulting in a decrease of Δλ with the increase in temperature. Initially, the molecules are in a uniformly distributed state, and it is difficult to show significant changes by adding low temperatures, while adding high temperatures causes the molecules to diffuse too quickly and lead to aggregation. Moreover, the optimal temperature of 40 °C is relatively low in the experimental group of conditions, and the changes in the case of less than 40 °C are not as pronounced as in the case of high temperatures.

#### 3.5.2. UV Intensity on the Reflectance Bandwidth of Samples

**Figure 8:**
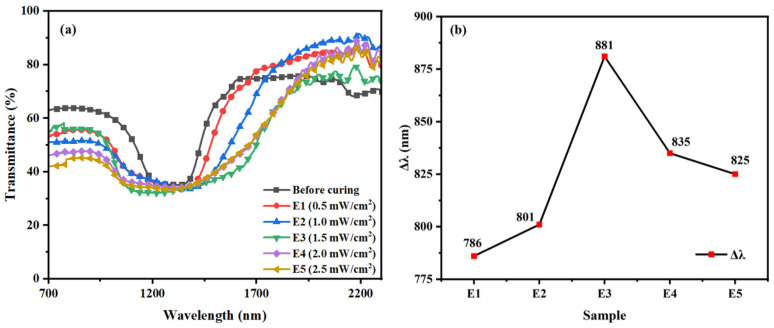
Group E samples: Trends of (**a**) transmission spectra and (**b**) Δλ with UV intensity.

For Group E samples in Table 1, they were placed under different UV intensities (0.5 mW/cm^2^, 1.0 mW/cm^2^, 1.5 mW/cm^2^, 2.0 mW/cm^2^, 2.5 mW/cm^2^) at 40 °C for 20 min for polymerization and were named as E1, E2, E3, E4, and E5, respectively. Figure 8 shows the transmission spectra and the corresponding Δλ of the samples (E1–E5) after polymerization. As can be seen in Figure 8b, there is a trend of increasing and then decreasing Δλ. It increases from 786 nm to 881 nm and then decreases from 881 nm to 825 nm. Δλ reaches its maximum at the light intensity of 1.5 mW/cm^2^. When the UV intensity is small (0.5 mW/cm^2^ to 1.5 mW/cm^2^), increasing the UV intensity will promote the diffusion of UV-absorbing dyes can form a greater gradient of UV intensity in the direction of the thickness of the liquid crystal films, and the polymerization rate of the polymerizable monomer RM257 is accelerated, and it will diffuse to the near-light side at a faster rate. Similarly, the chiral compound R5011 will move to the far-light side at a faster rate, which makes it easier to induce the pitch gradient distribution and broaden the reflection bandwidth of the samples. When the UV intensity is stronger (1.5 mW/cm^2^ to 2.5 mW/cm^2^), the polymerizable monomer RM257 will be rapidly polymerized at a larger polymerization rate to form a more compact polymer network, which means that the UV-absorbing dyes and R5011 cannot be completely diffused, and it is not easy to form the gradient distribution of the pitch, so that the Δλ of the samples shows a decreasing tendency with the increase in UV intensity.

#### 3.5.3. Polymerization Time on the Reflectance Bandwidth of Samples

**Figure 9:**
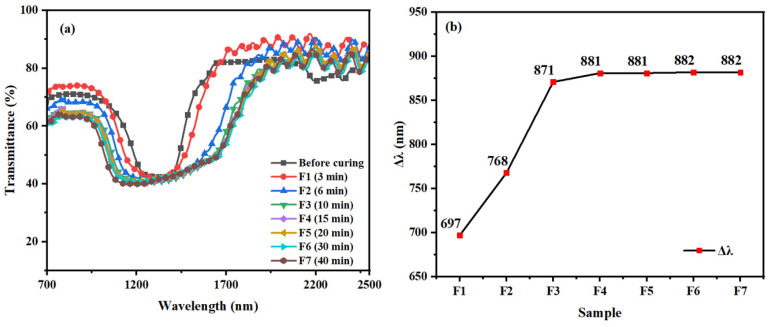
Group E samples: Trends of (**a**) transmission spectra and (**b**) Δλ with polymerization time.

For the samples of group F, they were placed under 1.5 mW/cm^2^ UV irradiation, the polymerization temperature was controlled at 40 °C, and the polymerization reactions were carried out at different times (3 min, 6 min, 10 min, 15 min, 20 min, 30 min, and 40 min), which were named F1, F2, F3, F4, F5, F6, and F7, respectively. Figure 9 shows the transmission spectra of samples (F1–F7) after polymerization and the corresponding Δλ. The Δλ of samples (F1–F5) rises from 697 nm to 881 nm during the first 20 min of polymerization. After 20 min of polymerization, Δλ remains almost constant at approximately 881 nm, indicating substantial polymerization of the polymerized monomers. In this liquid crystal system, UV irradiation affects the diffusion of the organic dye UV327 and the inorganic dye ZnO NPs, as well as the diffusion of the polymerizable monomer RM257 and the chiral molecule R5011, and the degree of monomer polymerization. During the first 20 min of the reaction, light absorption is promoted, and a UV gradient is formed by the diffusion of the organic dye UV327 and the inorganic dye ZnO NPs. At the same time, the polymerizable monomer RM257 and the chiral molecule R5011 begin to diffuse, and the polymer network is gradually formed due to the polymerization of RM257. As a result, Δλ increases rapidly during the first 20 min. After 10 min of irradiation, the polymer network is almost completely formed, leading to a significant slowdown in the diffusion of the chiral molecule R5011. Consequently, Δλ hardly increases anymore, indicating that the polymerization is complete after 20 min.

### 3.6. Optimal Sample Comparison

**Figure 10:**
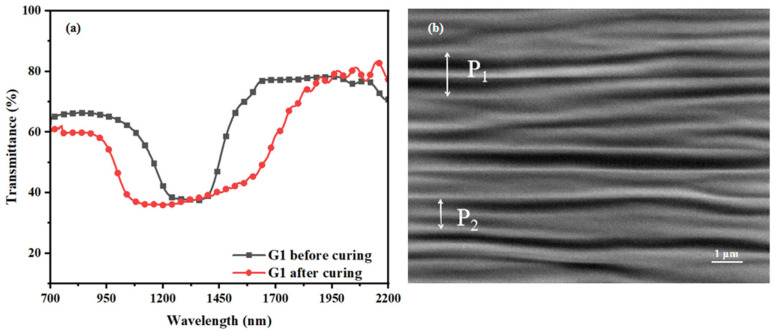
Optimal sample G1: Transmission spectra (**a**) and SEM image of the optimal sample G1 after curing (**b**); the arrows show the pitch.

According to the optimal content and experimental conditions determined in the above experiments, sample G1 (BHR32100-100/RM257/R5011/IRG-651/UV327/ZnO NPs 86.40/12.00/1.00/0.30/0.12/0.18) was prepared and polymerized for 20 min at a UV intensity of 1.5 mW/cm^2^, and the polymerization temperature was kept at 40 °C. As can be seen from the transmission spectra of sample G1 before and after polymerization in Figure 10a, the Δλ of sample G1 was broadened to 881 nm after the completion of polymerization. It demonstrates that the method of preparing broadband reflective cholesteric liquid crystal films using the hybrid method of organic and inorganic UV-absorbing dyes is capable of achieving significant broadening of the Δλ. The morphology of the film was analyzed using SEM, as shown in Figure 10b. It is obvious from the figure that pitch P_1_ > P_2_, which means that a pitch gradient distribution is formed in the direction of film thickness after the polymerization is completed, indirectly proving that the broadening of Δλ is caused by the pitch gradient distribution.

## 4. Conclusions

In this work, we successfully prepared cholesteric liquid crystal broadband reflective films doped with a hybrid dye consisting mainly of inorganic zinc oxide nanoparticles and the organic dye UV-327. This method effectively solves the problem of poor dispersion of ZnO NPs in organic systems. The characterization using polarization microscopy and ultraviolet transmission spectroscopy shows that the addition of UV327 to ZnO NPs significantly improves their dispersion and broadens the bandwidth of the films. In addition, we investigated the effects of various factors, including the compositional concentration of the polymerizable monomer RM257, the reaction temperature, the intensity of UV irradiation, and the reaction time, on the Δλ. Through these explorations, we determined the optimal reaction conditions under which the UV transmission spectroscopy indicated the reflection broadband (Δλ) of 881 nm for this best sample, proving its excellent performance. It provides a new idea for the future preparation of CLC films with broadband reflective properties, adding to their applications in energy-efficient buildings, smart windows, or coatings.

## Figures and Tables

**Table 1:** Sample group allocation ratios.

Sample Number	BHR32100-100/RM257/R5011/IRG-651/UV327/ZnO NPs (wt%)
A1	86.40/12.00/1.00/0.30/0.30/0.00
A2	86.40/12.00/1.00/0.30/0.24/0.06
A3	86.40/12.00/1.00/0.30/0.18/0.12
A4	86.40/12.00/1.00/0.30/0.12/0.18
A5	86.40/12.00/1.00/0.30/0.06/0.24
A6	86.40/12.00/1.00/0.30/0.00/0.30
B1	90.40/8.00/1.00/0.30/0.12/0.18
B2	88.40/10.00/1.00/0.30/0.12/0.18
B3	86.40/12.00/1.00/0.30/0.12/0.18
B4	84.40/14.00/1.00/0.30/0.12/0.18
B5	82.40/16.00/1.00/0.30/0.12/0.18
C1	86.50/12.00/0.90/0.30/0.12/0.18
C2	86.40/12.00/1.00/0.30/0.12/0.18
C3	86.30/12.00/1.10/0.30/0.12/0.18
C4	86.20/12.00/1.20/0.30/0.12/0.18
C5	86.10/12.00/1.30/0.30/0.12/0.18
D1	86.40/12.00/1.00/0.30/0.12/0.18
D2	86.40/12.00/1.00/0.30/0.12/0.18
D3	86.40/12.00/1.00/0.30/0.12/0.18
D4	86.40/12.00/1.00/0.30/0.12/0.18
D5	86.40/12.00/1.00/0.30/0.12/0.18
E1	86.40/12.00/1.00/0.30/0.12/0.18
E2	86.40/12.00/1.00/0.30/0.12/0.18
E3	86.40/12.00/1.00/0.30/0.12/0.18
E4	86.40/12.00/1.00/0.30/0.12/0.18
E5	86.40/12.00/1.00/0.30/0.12/0.18
F1	86.40/12.00/1.00/0.30/0.12/0.18
F2	86.40/12.00/1.00/0.30/0.12/0.18
F3	86.40/12.00/1.00/0.30/0.12/0.18
F4	86.40/12.00/1.00/0.30/0.12/0.18
F5	86.40/12.00/1.00/0.30/0.12/0.18
F6	86.40/12.00/1.00/0.30/0.12/0.18
F7	86.40/12.00/1.00/0.30/0.12/0.18
G1	86.40/12.00/1.00/0.30/0.12/0.18

## Data Availability

Data are contained within the article.
